# Acute Thrombosis of Left Anterior Descending Coronary Artery During Anaphylactic Reaction to Contrast Dye

**DOI:** 10.7759/cureus.23679

**Published:** 2022-03-31

**Authors:** John Cox, Cali Clark, Zachary Anderson

**Affiliations:** 1 Cardiology, Freeman Health System, Joplin, USA; 2 Internal Medicine, Freeman Health System, Joplin, USA; 3 College of Osteopathic Medicine, Kansas City University of Medicine and Biosciences, Joplin, USA

**Keywords:** intravenous contrast dye, anaphylactic shock, left anterior descending coronary artery, acute arterial thrombosis, anaphylaxis

## Abstract

Anaphylaxis is a systemic inflammatory response to an antigen and can result in hemodynamic compromise. While uncommon, it remains an important differential diagnosis in the setting of intraprocedural hypotension. Acute thrombosis has been associated with anaphylaxis and should be suspected based on clinical symptoms. We describe a clinical case of intraprocedural anaphylaxis secondary to intravenous contrast dye leading to hypotension and acute thrombosis of the left anterior descending coronary artery.

## Introduction

As the use of intravenous (IV) contrast dye becomes more widespread, it has become more uncommon for acute anaphylaxis due to contrast to be observed in the cardiac catheterization lab. However, this should be included in the differential diagnosis of acute hypotension during the procedure, and early administration of epinephrine is vital. While the role of thrombosis in anaphylaxis has not been extensively studied, it may be necessary to evaluate if this has occurred based on a patient’s clinical presentation. Additionally, patients should be closely monitored for 24 hours afterward in a critical care setting for delayed or biphasic reactions, which could compromise the patient’s airway or lead to hemodynamic instability.

## Case presentation

The patient is a 64-year-old man who presented with escalating exertional chest pain radiating down his left arm and into his jaw with exertion over the last 4 days. On physical examination, the patient was in no acute distress and had a regular rate and rhythm. Laboratory test results were significant for elevated troponin I at 0.617 ng/mL (0.012-0.120 ng/mL) and a hemoglobin A1c of 9.14%. The patient’s past medical history was significant for hypertension, hyperlipidemia, diabetes mellitus, tobacco use disorder, and a family history of coronary artery disease in his mother and father. The patient was transported to our facility’s cardiac catheterization lab and coronary angiography was performed via the right radial artery, revealing a 90% occlusion of the left anterior descending artery at the origin of the first diagonal branch, with a diffuse disease of 50-60% above and below the stenosis. This was identified as the culprit lesion for the patient’s non-ST elevation myocardial infarction.

**Figure 1:**
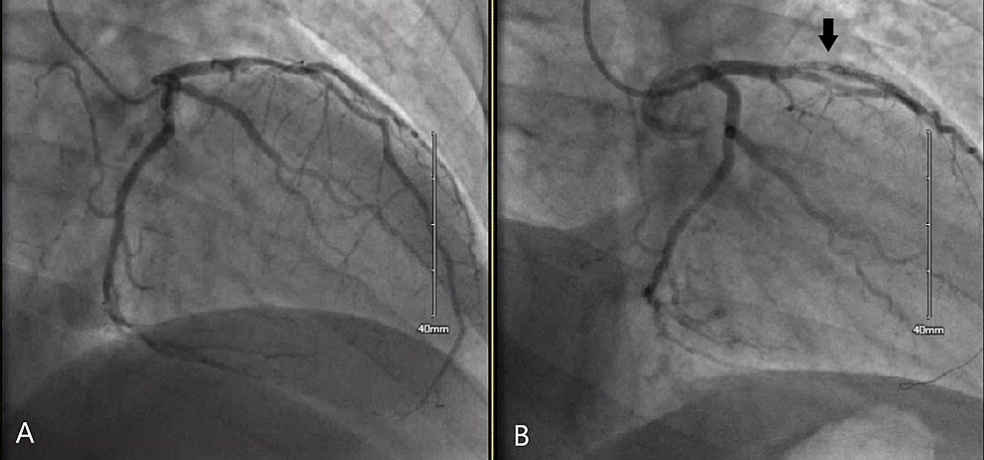
Coronary angiogram showing initial image of left coronary arteries (Panel A) and later image of LAD artery with diffuse thrombosis (Panel B). LAD: left anterior descending

A wire was advanced down the left anterior descending (LAD) and a 3.0 × 20 mm stent was deployed with a resolution of the stenosis. Immediately afterward, the patient became markedly hypotensive with a blood pressure of 64/39. The wire was retrieved from the left main coronary artery. Multiple vasopressor medications were administered, including phenylephrine, norepinephrine, and epinephrine, with minimal resolution of the hypotension. A bedside echocardiogram was obtained which showed preserved left ventricular function and no pericardial effusion. Repeat coronary angiography showed diffuse thrombosis of the LAD and diagonal artery, as seen in Figure 1B. At this time, it was suspected that the hypotension was most likely from an anaphylactic reaction to the IV contrast dye, Isovue 300. As the patient had already been administered IV epinephrine along with two other vasopressors, he was given diphenhydramine, famotidine, and methylprednisolone. Blood pressure recovery occurred rapidly afterward despite the ongoing coronary thrombosis. Clot extraction was performed, along with injection of intracoronary tissue plasminogen activator and IV cangrelor. Additional stenoses were noted at the proximal and distal portions of the first stent, and two additional stents were deployed on either side with good results.

The patient was kept overnight in the critical care unit and monitored for any delayed reaction. Vital signs remained stable, however, he did develop mild angioedema within the first few hours after the event, which resolved without airway compromise. Serum tryptase was elevated at 59.5 ng/mL (<11.0), confirming an anaphylactic reaction. He was observed on the cardiac floor for an additional day and then discharged home. At his cardiology clinic follow-up appointment, the patient reported no further symptoms.

## Discussion

Anaphylaxis is a severe systemic inflammatory response to an antigen leading to life-threatening airway and circulation difficulties and must be recognized and treated rapidly [1]. These patients require close monitoring in a critical care setting for delayed or biphasic reactions that could lead to hemodynamic compromise. As the use of IV contrast dye is increasingly more widespread, many patients become aware of their anaphylactic reaction before reaching the cardiac catheterization lab. Therefore, it is less common for the initial presentation of acute anaphylaxis to occur during this procedure, yet it remains an important differential diagnosis for intraprocedural hypotension.

While thrombosis is an important part of many inflammatory reactions, its role in anaphylaxis is not well established. Virchow’s triad has been used to illustrate the pathophysiology behind thrombosis and includes endothelial injury, hypercoagulable state, and stasis of blood flow. Coronary artery disease and atherosclerotic plaques contribute to endothelial disruption and damage and lead to impaired blood flow patterns across the lesions. Inflammation plays an important role in coronary artery disease, diabetes mellitus, and tobacco abuse disorder, and leads to a hypercoagulable state [2].

Anaphylaxis contributes to a hypercoagulable state due to the release of many factors and leads to stasis of blood flow secondary to severe vasodilation and hypotension. Anaphylactic events are characterized by a release of multiple mediators, including IgE antibodies, complement, mast cells, and histamine. Recent literature has highlighted the role of platelet-activating factor during this process, which is known to increase platelet aggregation and may contribute to thrombosis formation [3].

Management of anaphylaxis and associated distributive shock requires first ensuring the patient’s airway is secure, as severe angioedema can quickly lead to airway obstruction and compromise. Early epinephrine administration is vital as delayed administration is associated with poor outcomes and death. Intramuscular injection is preferred over subcutaneous, and IV can be considered when patients are very hypotensive or in shock. Supplemental oxygen and aggressive fluid resuscitation should also be given. If the hypotension is refractory to these measures, additional vasopressors should be started to maintain mean arterial pressure. Current literature supports the use of adjunctive therapies to epinephrine for symptom management, but not as treatments for airway compromise, hypotension, or shock. Patient’s should be monitored in a critical care setting after an anaphylactic reaction for 24 hours in case a delayed or biphasic reaction occurs [4].

## Conclusions

While there are many causes of intraprocedural hypotension, anaphylaxis remains an important differential diagnosis that must be considered as it can lead to shock and death if not recognized and treated rapidly. Management of anaphylaxis includes early administration of epinephrine, addition of secondary vasopressors if needed, and adjunctive treatments for symptom management. Recent literature has illustrated a correlation between anaphylaxis and thrombosis, including the role of mediators such as platelet-activating factors, however, further research needs to be conducted to define the relationship and clinical consequences.
